# Sarcomeric network analysis of ex vivo cultivated human atrial appendage tissue using super-resolution microscopy

**DOI:** 10.1038/s41598-023-39962-1

**Published:** 2023-08-10

**Authors:** Oleksandra Chabanovska, Heiko Lemcke, Hermann Lang, Brigitte Vollmar, Pascal M. Dohmen, Robert David, Christian Etz, Catharina Neßelmann

**Affiliations:** 1 Reference and Translation Center for Cardiac Stem Cell therapy (RTC), Department of Cardiac Surgery, Rostock University Medical Center, 18057 Rostock, Germany; 2 Department of Life, Light, and Matter of the Interdisciplinary Faculty, Rostock University, 18059 Rostock, Germany; 3 Department of Operative Dentistry and Periodontology, Rostock University Medical Center, 18059 Rostock, Germany; 4 Rudolf-Zenker-Institute of Experimental Surgery, Rostock University Medical Center, 18059 Rostock, Germany; 5 Department of Cardiac Surgery, Rostock University Medical Center, 18059 Rostock, Germany; 6 Department of Cardiothoracic Surgery, Faculty of Health Science, University of the Free State, Bloemfontein, 9301 South Africa

**Keywords:** Cytoskeleton, Super-resolution microscopy

## Abstract

Investigating native human cardiac tissue with preserved 3D macro- and microarchitecture is fundamental for clinical and basic research. Unfortunately, the low accessibility of the human myocardium continues to limit scientific progress. To overcome this issue, utilizing atrial appendages of the human heart may become highly beneficial. Atrial appendages are often removed during open-heart surgery and can be preserved ex vivo as living tissue with varying durability depending on the culture method. In this study, we prepared living thin myocardial slices from left atrial appendages that were cultured using an air-liquid interface system for overall 10 days. Metabolic activity of the cultured slices was assessed using a conventional methyl thiazolyl tetrazolium (MTT) assay. To monitor the structural integrity of cardiomyocytes within the tissue, we implemented our recently described super-resolution microscopy approach that allows both qualitative and quantitative in-depth evaluation of sarcomere network based on parameters such as overall sarcomere content, filament size and orientation. Additionally, expression of mRNAs coding for key structural and functional proteins was analyzed by real-time reverse transcription polymerase chain reaction (qRT-PCR). Our findings demonstrate highly significant disassembly of contractile apparatus represented by degradation of $\alpha$-actinin filaments detected after three days in culture, while metabolic activity was constantly rising and remained high for up to seven days. However, gene expression of crucial cardiac markers strongly decreased after the first day in culture indicating an early destructive response to ex vivo conditions. Therefore, we suggest static cultivation of living myocardial slices derived from left atrial appendage and prepared according to our protocol only for short-termed experiments (e.g. medicinal drug testing), while introduction of electro-mechanical stimulation protocols may offer the possibility for long-term integrity of such constructs.

## Introduction

The understanding of cardiac-specific cellular physiology and pathology largely relies on the biological models that are used to recreate the basic physiological or impaired clinical condition. The most reliable model for basic and therapeutic cardiovascular research is represented by human cardiac tissue. Unfortunately, the availability of human heart biopsies is strongly limited. To this account, the cavital regions of the left and right atria of the human heart, also known as the atrial appendages could serve as an accessible source of human atrial tissue.

Both left and right atrial appendages (LAA and RAA, respectively) derive from primary atrium^1^ and show different characteristics in terms of anatomy, physiology, pathophysiology, molecular profile and clinical implications. The gross anatomy of the left atrial appendage is a finger-like shape with a small entrance and a tubular lumen in contrast to the RAA showing a pyramidal figure with a wide entrance. Due to their primordial origin, both atrial appendages show a characteristic rough endocardium formed from the basal mesodermal layer as well as distinctive trabeculation rich in pectinate muscles. The pectinate muscles in the RAA are all connected to each other and form a “dendritic” network, while in the LAA the muscles are running in parallel and show less pronounced connections^2^. Physiologically, the appendages predominantly serve as a contractile reservoir performing suction during systole, while acting as decompression chamber conduit in diastolic phase^3^.

The atrial appendages also function as an endocrine organ by producing atrial natriuretic peptides^4^. These peptides display several biological effects and play an important role as biomarkers in heart failure, atrial fibrillation, acute coronary syndrome, myocarditis and other cardiac conditions such as sepsis or rejection after heart transplant^5^. Furthermore, Leinonen et al. (2013) revealed that LAA contains up to 20% of endogenous cardiac progenitor cells. This finding indicates a potentially supporting role of LAA in cardiac regeneration^6^.

Generally, the left atrial appendage has a higher clinical relevance than its right counterpart. Based on 3D imaging data, LAA has a more complex morphology, higher surface to volume ratio as well as higher amount of pectinate muscles than RAA^2^. In case of atrial fibrillation (AF), these factors strongly favor the risk of thrombus formation in LAA leading to thromboembolism. Indeed, thrombotic events in diagnosed AF cases are less common in RAA^7^.

Both atrial appendages can be harvested during cardiac surgery when atrial appendage occlusion is planned. The LAA occlusion is recommended for stroke prevention in patients with atrial fibrillation undergoing cardiac surgery (Class II b recommendation, 2020 ESC guidelines for the diagnosis and management of atrial fibrillation). In fact, it was shown in a randomized, multi-center trial that the simultaneous surgical occlusion of the LAA in patients with atrial fibrillation undergoing cardiac surgery leads to a significantly lower risk of ischemic stroke or systemic embolism^8^. However, Dominguez et al. (2018) indicated that LAA resection rathern than occlusion would effectively reduce risks of stroke^9^. Hence, it is to expect that the surgical exclusion of LAA will increasingly become the preventative therapy choice for AF patients with high risk of thromboembolic occurrence or in case of contraindication for anticoagulation therapy. This trend would also open up an excellent opportunity for cardiac researchers by providing this unique human heart tissue of atrial origin, which is otherwise rather problematic to obtain.

The removed atrial tissue can be maintained in an ex vivo culture system as living ultrathin (200–400 μm) myocardial slices. Such samples are highly beneficial since they accurately reflect the native heart architecture including a variety of unique cardiac-specific resident cells such as cardiomyocytes, fibroblasts, endothelial and inflammatory cells. Therefore, a wide range of research areas can be covered, e.g. patient-related assessment of cardiac tissue physiology or drug-induced cardiotoxicity^10–12^, cardiac fibrosis research^13,14^, investigation of inter-cellular communications^15^ or exploration of cardiac regeneration approaches^16,17^. Recently, Zhou et al. (2022) published a highly efficient protocol for isolation and culture of primary cardiomyocytes from human LAA slices. This study demonstrated remarkable cardiac functionality and pronounced response to cardiotoxic drugs in isolated primary cardiomyocytes in contrast to the artificial human induced pluripotent stem cell (hiPSC)-derived cardiomyocytes (CMs), which responses were less distinct^18^. Although hiPSC-CMs represent a popular in vitro cardiac model, these cells clearly lack maturity. Regarding this aspect, our group recently introduced a novel super-resolution microscopic approach to monitor the maturation of hiPSC-CMs by studying the cellular organization of the sarcomere network^19^. Herein, we revealed that the immature status of human iPSC-CMs emerges from a yet underdeveloped contraction apparatus and is similar to the morphology found in murine neonatal cardiomyocytes. Unfortunately, although this work also provides analysis of adult cardiomyocytes isolated from mouse hearts, the study does not involve analysis of primary human cells.

Here, we proceed the exploration of cardiac sarcomere network initiated by Skorska et al. by utilizing our group‘s super-resolution microscopy technique^19^ on human adult cardiomyocytes. Super-resolution microscopy allowed us to overcome the diffraction-based resolution limit of 220 nm, leading to more accurate data sets on sarcomere integrity. In particular, we applied structured illumination microscopy, established by Gustafsson et al^20^, that enables two times better resolution compared to conventional confocal imaging. The projection of a grid-line illumination pattern on a microscopic specimen results in moiré fringes, containing information of sub resolution structures. For this analysis, we adapted a previously described protocol for vibratome-cut ventricular samples^21^ and prepared comparable slices of excluded left atrial appendage. Currently, the golden standard in the field of *in vitro* culture systems for cardiac slices necessary includes a permanent electromechanical stimulation (EMS) of the tissue in order to ensure a prolonged (more than five weeks) maintenance of functionality^22,23^. Prior to EMS systems, the cultivation techniques largely relied on an air-liquid interface (ALI) method using semi-porous culture inserts without any additional stimuli. ALI cultivation allows a nutritional flow from the culture medium throughout the permeable membrane to the bottom side of the resting slice. Simultaneously, oxygen from the air supplies the top side of the slice. However, in the absence of physiologically essential electrical stimulation as well as mechanical preload, the slices rapidly lose their contractile function, alter the initial morphology and expand the extracellular space^23^. The ALI culture of human slices derived from ventricular or atrial free wall was previously reported by several research groups^24–26^. However, durability of LAA slices via ALI cultivation was not explored before. Taking into account our aim that is to transfer our super-resolution based approach to human ex vivo cultivated heart tissue and highlight its feasibility as well as reliability in a human cardiac model, we chose the ALI system to delineate the sarcomere disassembly in a time-efficient manner. In contrast, a long-term stimulated cultivation would delay the aims of the present study since EMS acts highly protective of sarcomere units. Our experimental data demonstrate that despite the relatively high metabolic activity lasting up to 10 days in ALI-cultured tissue, a highly accelerated structural decay of sarcomere network starting from third day of cultivation could be observed. This evidence sharply underlines the low durability of the ALI-cultured cardiac tissue and thus limits its application to short-term experiments. However, this first human ex vivo sarcomere analysis using super-resolution microscopy could be further applied for an evaluation of the tissue under different culture conditions, e.g. EMS systems or pharmacological treatments. Moreover, the presented results are useful as a reference for structural maturity and integrity of human stem cell-derived cardiomyocytes.

## Results

### Tissue metabolic activity

**Figure 1:**
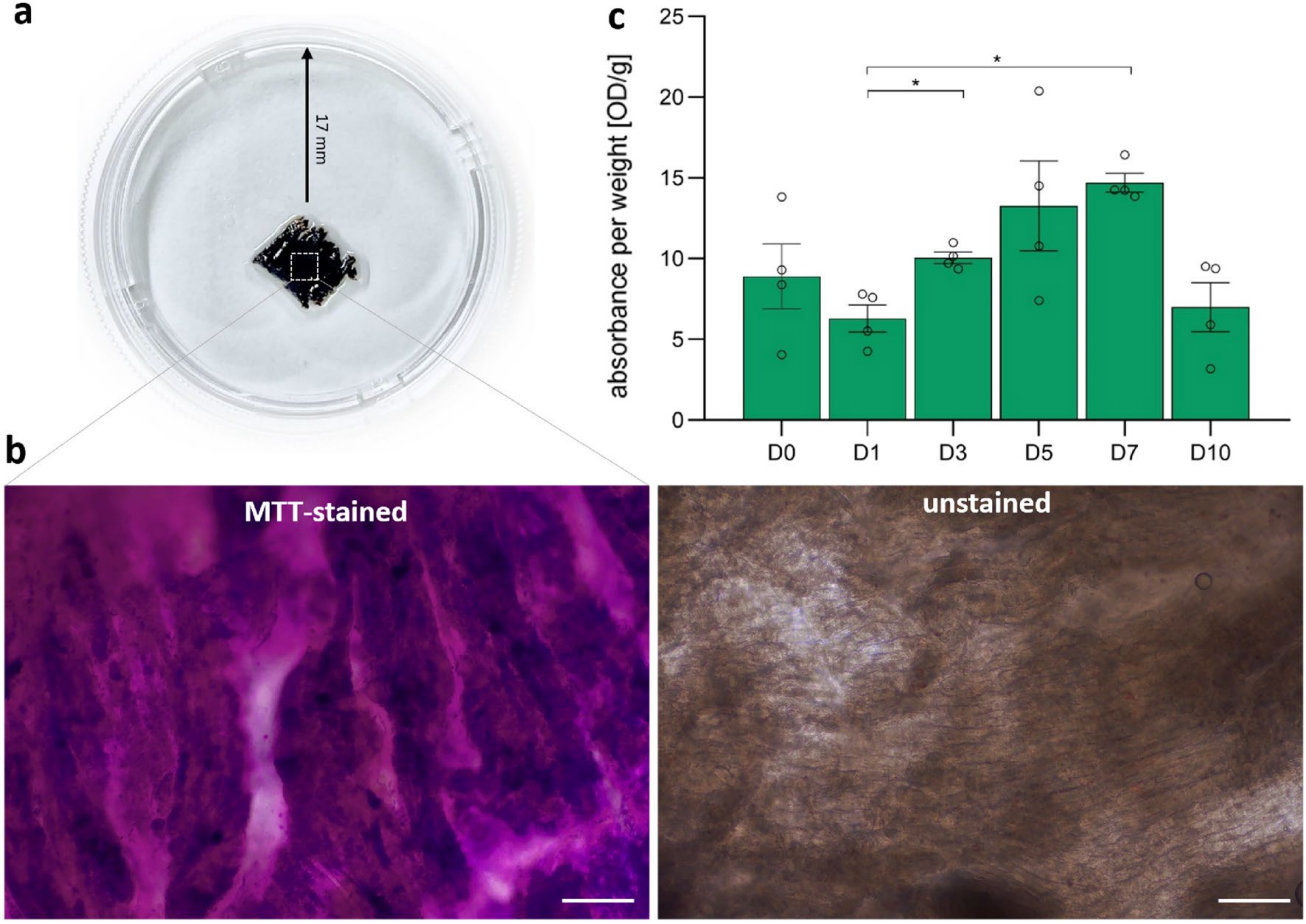
Time-dependent analysis of cellular metabolic activity in LAA slices using MTT assay. (**a**) Representative image of a LAA slice in a petri dish that actively metabolized the applied MTT salt into formazan, thereby causing a dark violet appearance of the tissue. (**b**) Representative enlarged bright-field microscopic image of LAA slice illustrates accumulated formazan in highly viable MTT-stained (left) vs. unstained (right) tissue. Scale bar = 100 μm. (**c**) The metabolic activity of LAA slices obtained from multiple patients was evaluated at six different time points. Using spectrophotometric measurement, the optical absorbance of dissolved formazan was defined for each slice and normalized to its weight. The analysis indicated a constantly rising metabolic activity within the first week of cultivation, which promptly dropped thereafter. Statistical significance was determined using one-way ANOVA followed by Tukey post hoc test, n = 4 slices (from 4 independent LAA samples) for each time point, *p $< 0.05$.

The metabolic activity of prepared slices was determined over a period of 10 days. For this purpose, we used the methyl thiazolyl tetrazolium (MTT) assay. Here, the soluble colorless tetrazolium salt is metabolized to the insoluble purple formazan by NAD(P)H-dependent cellular oxidoreductase enzyme. The resulting formazan is retained in the cytoplasm of living cells (Fig. 1a). Figure 1b additionally illustrates mostly regular arrangement of cardiomyocytes in fresh unstained LAA slices, while an enlarged image of a MTT-stained slice represents the high formazan content within cells indicating high viability. Formazan can be subsequently retrieved by a solvent reagent. The final solution is analyzed using a spectrophotometer. The measured optical density degree represents the concentration of the initially metabolized salt and therefore indicates metabolic activity of the tissue. Our analysis revealed a 30% decline in metabolic activity after the first 24 h of cultivation compared to freshly prepared slices (D0 vs. D1: 8.89 ± 2.00 vs. 6.29 ± 0.85) (Fig. 1c). However, the cultivated tissue gained a significant metabolic boost (*p* = 0.03) in the next two days of cultivation (D3: 10.05 ± 0.34), thereby exceeding the activity of fresh samples by 13%. The tissue metabolism demonstrated further significant reinforcement (*p* = 0.04) and reached its peak at the end of the first week (D5 vs. D7: 13.27 ± 2.78 vs. 14.70 ± 0.58). A strong 50% drop was observed thereafter at D10 (6.99 ± 1.52) that marked the progressive tissue disintegration.

**Figure 2:**
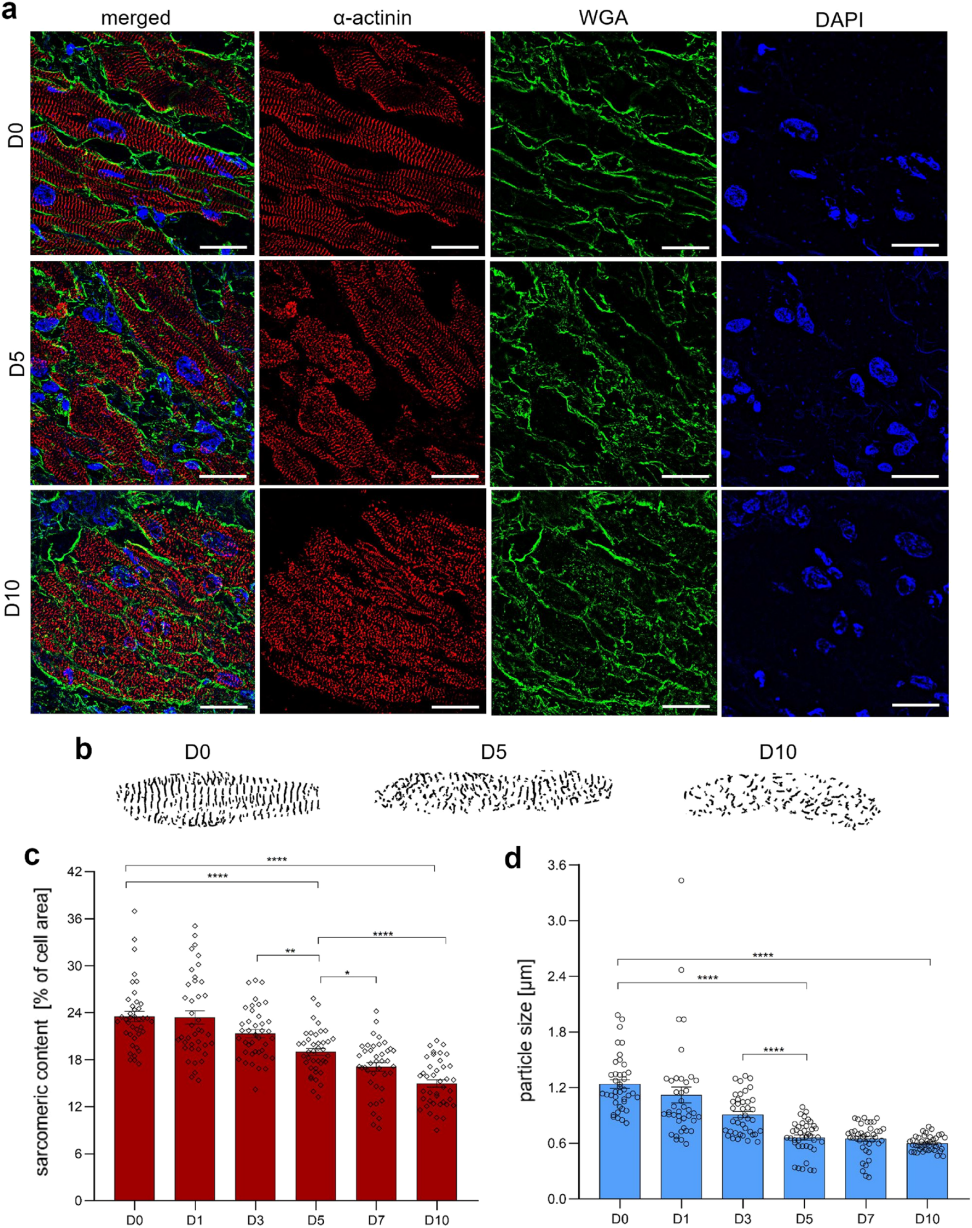
Time-dependent SIM-based analysis of sarcomeric network in LAA cardiomyocytes. (**a**) Panels demonstrate representative SIM images of LAA cryosections immunolabeled at D0, D5 and D10 with an antibody directed against $\alpha$-actinin (red), WGA conjugate to visualize cell membrane (green), DAPI staining for cell nuclei (blue) as well as the overlay of all three stainings (merged). Fluorescence images show clear disorganization of $\alpha$-actinin in cardiomyocytes over the course of cultivation. Scale bar = 20 μm. (**b**) Binary images of the $\alpha$-actinin network illustrate the extent of degradation. The images were adjusted for better visualization and do not represent the actual cell size. (**c**) The qualitative evaluation was supported by a quantitative analysis of the mean sarcomere density in cardiomyocytes that revealed a significant constant loss of $\alpha$-actinin content over time. (**d**) The continuing cultivation of LAA slices resulted in progressing utilization of $\alpha$-actinin particles leading to a highly significant reduction of its size. Statistical analysis of quantitative data was performed using one-way ANOVA followed by Tukey post hoc test, n = 40 cells (10 cells per slice; 4 slices from 4 independent LAA samples) for each time point, *p $< 0.05$, **p $< 0.01$, ***p $< 0.001$, ****$\textit{p} < 0.0001$.

**Figure 3:**
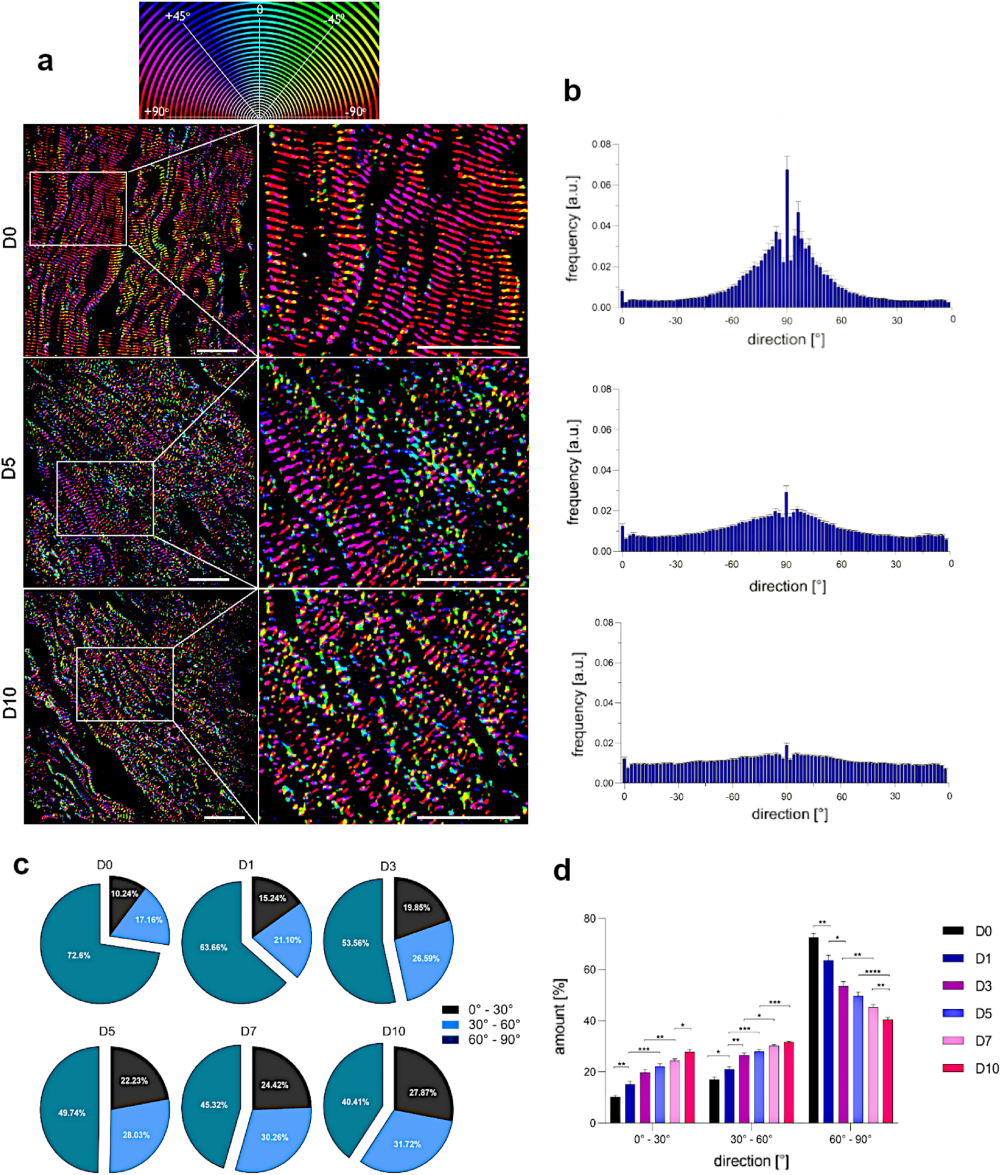
Time-dependent SIM-based evaluation of sarcomere orientation in LAA cardiomyocytes. (**a**) Representative SIM images of antibody-labeled $\alpha$-actinin filaments taken at D0, D5 and D10 served as a source for qualitative orientation analysis. The direction of the sarcomeric network was colored according to the presented orientation map. The prevalent orientation in 70$^\circ$–90$^\circ$ is largely colored by red, pink and yellow. The multicolored sarcomere structures indicate high degree of disorientation. Scale bar = 20 μm. (**b**) Corresponding histograms demonstrate the mean distribution of $\alpha$-actinin direction that peaks between 75$^\circ$ and 90$^\circ$ in fresh slices but increasingly disperses over time. (**c**) Pie charts illustrate the time-dependent shift of filament direction in tissue cardiomyocytes from 60$^\circ$ - 90$^\circ$ to 0$^\circ$–60$^\circ$ indicating advancing disorientation. (**d**) A bar chart additionally emphasizes the highly significant substantial loss of the $\alpha$-actinin main direction throughout cultivation period. Statistical analysis of quantitative data was performed using two-way ANOVA followed by Tukey post hoc test, n = 40 cells (10 cells per slice; 4 slices from 4 independent LAA samples) for each time point, *p $< 0.05$, **p $< 0.01$, ***p $< 0.001$, ****p $< 0.0001$.

### Sarcomere network analysis

Next, time-dependent sarcomere organization of the cultured tissue was examined using super-resolution microscopy approach. Qualitative analysis of microscopic images revealed a progressive decay of $\alpha$-actinin filaments over time (Fig. 2a). While fresh slices (D0) demonstrated intact contractile apparatus, the disorganization became apparent at D5 and spread throughout the entire slice at D10. Binary images of representative cells that were stained against $\alpha$-actinin and analyzed before (D0), during (D5) and at the end (D10) of cultivation confirmed the extent of degradation (Fig. 2b). Furthermore, we quantified the sarcomere density that is given as a percentage of total $\alpha$-actinin structures per cell area (Fig. 2c). Our analysis indicated a consistent highly significant loss of $\alpha$-actinin filaments by 9–11% every second day of cultivation starting from D1 (D1 vs. D3 vs. D5 vs. D7: 23.40 ± 0.84 vs. 21.34 ± 0.51 vs. 19.00 ± 0.44 vs. 17.10 ± 0.47) and reaching its minimum at D10 (14.97 ± 0.47). We also assessed the integrity of $\alpha$-actinin filaments further referred as a particle size measured in μm (Fig. 2d). The incipient molecular disintegration rapidly increased during the first five days of cultivation and could be detected from early on. After the first day in culture, we observed 10% particle size reduction (D0 vs. D1: 1.24 ± 0.05 vs. 1.12 ± 0.09). The molecular dissociation became more distinct over the next two days and accounted for 20% filament decay from D1 to D3 (0.91 ± 0.03), whereas the most pronounced loss of structure (30%) was observed from D3 to D5 (0.66 ± 0.03; *p* $< 0.0001$). During the next five days, the decay decelerated. Finally, at D10 the measured particle size resulted in 0.60 ± 0.01 μm, which is 48% of the unimpaired filament as seen in uncultured slices at D0.

### Assessment of sarcomere orientation

**Figure 4:**
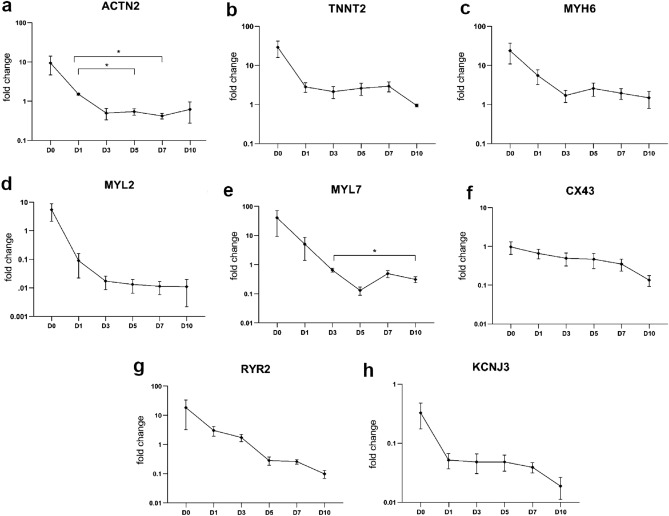
Relative expression of cardiac-specific structural and functional proteins in LAA cultivated over time analyzed by qRT-PCR. Levels of the transcripts encoded by (**a**) ACTN2, (**b**) TNNT2, (**c**) MYH6, (**d**) MYL7, (**e**) MYL2, (**f**) CX43, (**g**) RYR2 and (**h**) KCNJ3 genes were evaluated in cultured tissue at different time points. Except CX43, all targets demonstrated strongly decreased expression over time. Gene expression was normalized to the internal reference gene GAPDH in each sample and presented on a log scale as fold change. Statistical analysis of the data was performed using one-way ANOVA followed by Tukey post hoc test, n = 4 slices (from 4 independent LAA samples) per each time point, n = 3 slices (from 3 independent LAA samples) for D0 MYL7 and D10 TNNT2, CX43 and RYR2. *p $< 0.05$.

The contractile apparatus plays a major role for the functionality of cardiomyocytes. Here, the orientation of the structural fibers is essential to ensure proper force generation. Skorska et al. recorded that $\alpha$–actinin filaments in adult cardiomyocytes isolated from mouse are directed in 86.08$^\circ$ ± 0.69$^\circ$ to the longitudinal axis of the cell. Figure 4 summarizes our data on directionality of the $\alpha$-actinin filaments in human cardiomyocytes within a cultured tissue slice. The direction of filaments was visualized by assigning their orientation to a distinct color (Fig. 3a). This qualitative analysis demonstrated prevalently red, pink and yellow-colored filaments at D0 that corresponds with the main orientation between 70$^\circ$ and 90$^\circ$. Over time, the filaments became increasingly omnidirectional resulting in highly multicolored images. Therefore, the respective images are highly multicolored. These findings were confirmed by the corresponding histograms showing progressive dispersion of $\alpha$-actinin direction throughout the cultivation period (Fig. 3b). Furthermore, we classified three ranges of filament direction (0$^\circ$–30$^\circ$, 30$^\circ$–60$^\circ$ and 60$^\circ$–90$^\circ$) and calculated respective percentages. The quantification indicated that the vast majority (70%) of $\alpha$-actinin in fresh slices was directed between 60$^\circ$ and 90$^\circ$ (Fig. 3c). However, a significantly advancing disorientation of filaments was observed with every additional day in culture (Fig. 3d). Thus, only the half of the filaments had normal orientation at D5, which further reduced to 40% at D10.

### Gene expression analysis of mRNAs encoding structural and functional proteins

To investigate whether the observed morphological changes are also reflected on the gene expression level, we examined several cardiac-specific structural and functional markers that are essential for the contractile function using real-time reverse transcription polymerase chain reaction (qRT-PCR). mRNAs coding for sarcomere proteins like $\alpha$-actinin-2 (ACTN2), cardiac muscle troponin T (TNNT2), myosin heavy chain, $\alpha$ isoform (MYH6), myosin regulatory light chain 2, ventricular isoform (MYL2) as well as atrial isoform (MYL7) demonstrated a major decrease of the expression within the first 24 h of cultivation (Fig. 4a,e). Whereas the TNNT2 gene remained consistently suppressed starting from D1, the rest of the analyzed transcripts reached the minimum of expression at D3. We also evaluated the expression of a potassium voltage-gated channel Kir3.1 encoded by the gene KCNJ3 that plays important role for heartbeat regulation as well as ryanodine receptor 2 (RYR2) that is responsible for intracellular calcium handling (Fig. 4g,h). Both genes showed strongly reduced activity that progressively decreased over time. In contrast, the expression of connexin 43 (CX43), a crucial gap junction protein that participates in intercellular communication and ensures electrical impulse propagation, was relatively maintained within the first week in culture and began to decrease by D10 (Fig. 4f).

## Discussion

In this study, we demonstrate a reliable method to assess the structural integrity of ex vivo cultivated human atrial myocardium using super resolution-based microscopy. The described approach allows a comprehensive investigation of sarcomeric networks in tissue cardiomyocytes by determining total sarcomere content, $\alpha$-actinin orientation as well as filament size. Based on these essential parameters, our data propose reference values that indicate intact vs. decomposed contractile apparatus in cultured myocardium slices. Furthermore, we suggest the human LAA as a unique source of extremely valuable human cardiac tissue that can be maintained as thin slices in ALI culture for 3-4 days. We observed a significant reduction of $\alpha$-actinin content in tissue cardiomyocytes and strongly significant filament fragmentation after five days in culture, while metabolic activity was retained. In addition, we detected a strongly reduced mRNA levels coding for cardiac-specific structural and functional proteins after the first day in the culture indicating early suppression of the gene expression as a response to the ex vivo environment.

The preparation and cultivation of myocardial slices is a valuable method that has been utilized for decades to study various issues in the cardiovascular field. Since the release of the first protocols for in vitro-cultured heart tissue^27,28^, great advancements have been made regarding this technique. First, the heart slices became thinner (μm) due to improved slicing technology that allowed a reproducible precision cut^29,30^. In 2012, a semi-porous membrane was introduced as a uniform transwell insert that established the ALI culture, where thin human ventricular slices could be maintained for several weeks^21^. However, cardiac tissue requires electromechanical stimuli to sustain its contractile apparatus. Hence, in the non-physiological environment, the myocardial slices tend to lose > 90% of their contractility^13^. Therefore, novel approaches adopted mechanical preload and continuous electrical stimulation, which in combination successfully mimicked in vivo conditions. This combined method represents the most advanced technique for a long-term preservation of structural integrity and full functionality of the living cardiac slices^22,23^. Yet, for the purpose of monitoring and quantifying stages of $\alpha$-actinin decomposition as presented in our study, the ALI method enables much more time-efficient analysis since stimulated conditions delay the decay up to several months. Furthermore, transwell inserts for ALI cultures may be beneficial especially for short-term experiments (24–48 h). In contrast to EMS devices, which limit the range of parallel experiments by the availability of electrodes, ALI inserts enable a higher throughput and are more cost-effective in acquisition.

Until recently, animal hearts were the main source of cardiac tissue.Although cultivation of prepared slices derived from ventricular or atrial free-wall samples has been described before^24^, human heart biopsies are often challenging to obtain and therefore extremely rare. However, implementing explanted human atrial appendages as a source of cardiac tissue can resolve the problem of low accessibility of human heart material. The LAA is recommended for occlusion or exclusion to prevent embolic stroke and arrhythmias in patients suffering from atrial fibrillation, while RAA is routinely removed during open-heart surgery with cardiopulmonary bypass^2,31^. Moreover, utilizing the accessible human-derived primary tissue is still bears some advantages over *in vitro* iPSC-based approaches. Indeed, several studies reported on advanced 2D or 3D cardiac differentiation techniques that generate cells or cell constructs apparently resembling adult-like phenotype and physiological responses^32–34^. Yet, according to the latest reviews in this field^35–37^, the current *in vitro* models still require further optimization as the maturity of the final cell product as well as reproducibility of the utilized protocols are not yet fully guaranteed. Therefore, at present ex vivo human tissue still remains the most reliable cardiac model representing fully mature genuine myocardium. In 2022, Klumm et al. has described a highly efficient method for prolonged EMS-based cultivation of trabeculae isolated from RAA consisting largely from trabecular pectinate muscles^22^. Unfortunately, the tight elastic endocardium surrounding the inner muscle core makes it rather impractical to prepare thin slices from trabeculae. Therefore, the unsliced trabeculae require culture conditions with constant, intensive and frequent medium agitation to ensure adequate oxygen and nutrient supply to all layers of the tissue. In contrary, static ALI culture would not allow proper nutrient diffusion in tissues thicker than 400 μm. On the other hand, the area within the LAA entrance has lower trabeculation than in RAA. Depending on the LAA shape, the wall thickness around LAA orifice varies between   1.4–4.6 mm^38^. This particular area offers an opportunity to generate living LAA slices that can be cultured in both ESM-based and ALI-based systems. To our best knowledge, the present study is first to introduce the durability of cultured human LAA slices illustrating a short-term maintenance using straight-forward, commercially available transwell inserts.

First, we analyzed the metabolic activity of human LAA slices. This parameter was marked by an initial decline at D1, subsequent reinforcement from D3 to D7 followed by a repeated strong decrease observed at D10. Similar initial metabolic fluctuations had been described in a study by Thomas et al. (2016) cultivating human ventricular slices^25^. Here, the metabolic activity was measured using the MTT assay and varied between 20-25% within the first three days in culture. Recently, Boukhalfa et al. (2023) investigated the (MTT-based) viability of ALI-cultured canine ventricular slices in a time-dependent manner and presented a similar trend: an initial metabolic boost was detected at D3, which started to decline after a week in culture^26^. In contrast, Brandenburger et al. reported on relatively stable metabolism of sliced ventricular myocardium after four weeks in ALI culture compared to fresh control tissue. This discrepancy in durability may be attributed to the different microarchitecture of the atria and ventricles. Muscular fibers in ventricles show a regular, dense and homogenous alignment, while atrial myofibers are highly dispersed and more loosely aligned^39,40^. Furthermore, the connective tissue might play a crucial role in terms of metabolic turnover. For example, disease-free canine atrial fibroblasts demonstrated higher proliferation rate and higher abundance in the corresponding chamber compared to ventricular fibroblasts^41^. Of note, atrial fibroblasts isolated from LAA shared similar properties with fibroblasts derived from left atrial free wall. Another research work investigated healthy human atrial and ventricular fibroblasts and confirmed the higher proliferation activity of the former^42^. However, the present study recruited patients diagnosed with atrial fibrillation and did not include healthy donors. The accumulated evidence suggests that atrial fibrillation is the final manifestation of ongoing structural and electrical remodeling of the atria resulting from various conditions like aging, heart failure, hypertension, hypertrophy or diabetes mellitus^43^. These and other determinants commonly account for interstitial fibrosis that is highly associated with atrial fibrillation^44–46^. Although we did not perform a comparison between diseased and healthy LAA, we would expect a higher fibrotic content in our samples from AF patients. An increased content of highly proliferative fibroblasts could also contribute to the constantly rising metabolic activity of cultured LAA tissue that we observed during the first week.

On the other hand, the evaluation of sarcomere network indicated a major progressive decay and disorganization of contractile apparatus that started after the first 72 h in culture. At this point, the metabolic activity appeared slightly elevated compared to fresh slices. In 2018, the *Nomenclature Committee on Cell Death* stated that apoptotic cells retain an active metabolism and an intact cellular membrane until late-stage apoptosis^47^ Furthermore, we assume that the global disassembly of the $\alpha$-actinin network may be connected with metabolic rate due to increased activity of diverse proteolytic systems^48^. Recently, Russell and Sol’s (2021) reviewed mechanosignaling pathways in cardiomyocytes in context of optimized cellular energy consumption^49^. Pursuant to the article, the sarcomeric network is a dynamic structure that a cell can alter depending on needed performance. In order to match the energetic demands, gain of mass is imposed if greater contraction power is physiologically required. In the absence of mechanical stimuli, decreasing the mass is a favorable process in order to ensure the energy balance. This evidence supports our findings as we detected a vast decline of key transcripts encoding sarcomeric proteins (like $\alpha$-actinin, troponin T, myosin heavy and light chains) after one day in culture without any mechanical preload. In this case, structural realignment is likely to be initiated early at the genetic/RNA level and manifest at the protein level a few days later. The super-resolution microscopy-based approach described by Skroska et al. offers a detailed assessment of the contractile apparatus in tissue cardiomyocytes and can complement viability tests, especially when experiments evaluating contractility are not available. Moreover, our established approach could be generally useful to monitor sarcomere alterations: $\alpha$-actinin dis- and reassembly during cardiac mitosis^50^, infection-induced disarray of $\alpha$-actinin filaments^51^, proteolytic degradation of $\alpha$-actinin caused by myocardial ischemia^52^ or investigation of postmortem protein degradation patterns in forensic science^53,54^.

Taken together, we introduced a method for preparation and slicing of LAA that is a highly valuable source of human cardiac tissue. The ALI-based cultivation results in rapid tissue degradation that we monitored using recently described super-resolution microscopy. This technique allows a qualitative and quantitative evaluation of the sarcomere network and offers a reliable insight into disassembly processes in contractile apparatus occurring early within a cell. However, the parameter of metabolic activity of the tissue assessed using a conventional viability assay did not consider ongoing intercellular disorganization and, therefore, is not recommended for an early recognition of incipient tissue decay. Furthermore, our findings regarding human sarcomere network could be implemented as reference values for optimization of cardiac differentiation protocols in order to achieve a mature phenotype in human iPSC-derived cardiomyocytes. The short-term cultivation of LAA slices might be also advantageous for testing pharmacological compounds in order to examine the risks for acute cardiotoxicity. Furthermore, future optimization of electro-mechanical stimulation protocols may offer the possibility for long-term use of such constructs without losing their integrity.

### Limitations

Several limiting factors should be acknowledged. The obtained human tissues are derived from diseased patients sharing atrial fibrillation as a diagnosis that prescribes ablation of LAA. Therefore, it cannot be excluded that tissues harvested from healthy human hearts might demonstrate a different pattern of analyzed parameters. Furthermore, our ex vivo approach intentionally excludes electrical stimulation as this method drastically prolongs viability and tissue integrity. Instead, we present a novel microscopic method that quantifies the time-dependent stages of sarcomeric disassembly at a basal level. This application could be highly valuable for studies related to examination of sarcomeric integrity in cardiac tissues. Our study provides a thorough description of applied SIM technique as a central method to evaluate and monitor the structural state of the contractile apparatus. Although physiological assays were not a part of this report, we observed a spontaneous basal electrical activity in LAA slices within the first three days of cultivation (D0 and D3 in Supplementary Video S1 and S2 online, respectively) and following addition of MTT reagent at later time points (D5 in Supplementary Video S3 online). Future work will be needed to examine the electrical responses to diverse medical drugs.

## Methods

### Collection of atrial appendages

The procedure for tissue obtaining and its subsequent use for research purposes was approved by the Ethics Committee of the Medical Faculty at the University of Rostock (A 2018-0037). All patients gave informed written consent prior to surgery and sampling according to the Declaration of Helsinki. Surgical excision of the left atrial appendage was performed using a cut-and-sew method. This technique includes first the amputation of the LAA at the basis of the appendage by excision and then oversewing of the opening in multiple fashions (running/mattress sutures, single/double layer, with or without felt pledget reinforcement) by a non-absorbable suture. The removed LAA was immediately transferred into a tube containing cold modified Tyrode’s solution (MTS; in mM: 13.6 NaCl, 5.4 KCl, 0.9 CaCl_2_, 1 MgCl$_2$x6H$_2$O, 0.33 Na$_2$HPO$_4$, 30 2,3-butanedion monoxin, 5 HEPES; pH 7.4), placed on ice and brought into the research department. Material from 7 male patients in age between 54 and 76 with different indications for surgery was used for further analysis (Table 1).

**Table 1:** Characteristics of donors for left atrial appendage samples. All patients were diagnosed with atrial fibrillation as an indication for atrial appendage exclusion.

Patient number	Gender	Age	Diagnosis
1	Male	59	Mitral and tricuspid valve regurgitation
2	Male	54	Coronary artery disease
3	Male	61	Coronary artery disease
4	Male	76	Aortic and tricuspid valve regurgitation
5	Male	71	Aortic valve stenosis
6	Male	69	Aortic valve insufficiency and regurgitation
7	Male	56	Coronary artery disease

### Preparation and slicing of atrial appendages

**Figure 5:**
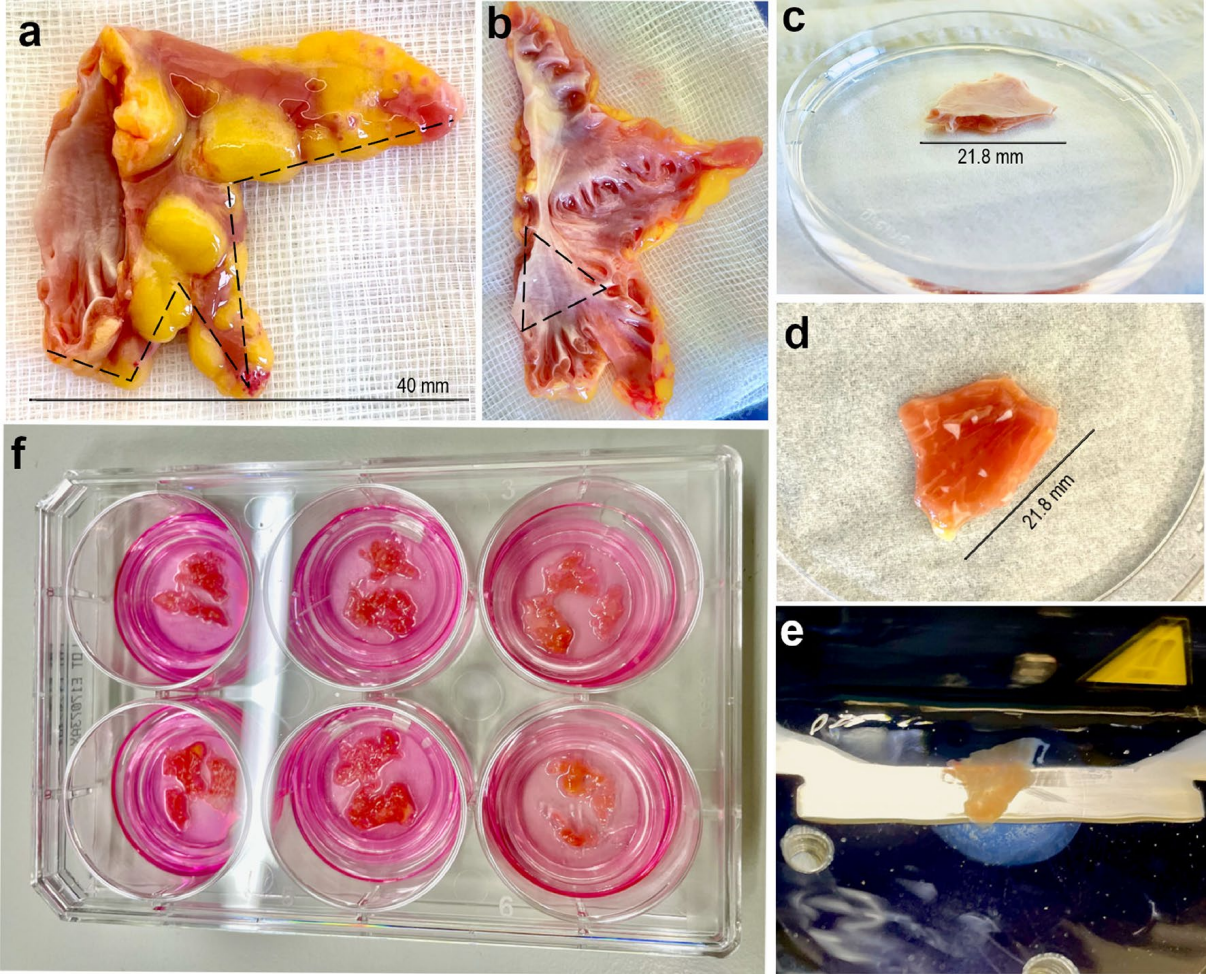
Preparation of left atrial appendage slices. (**a**) Representative image of excluded LAA that was cut open with scissors along the site marked by a dashed line. (**b**) The muscle-rich region in LAA with low trabeculation (marked by a dashed triangle) was used for further procedures. (**c, d**) A typical LAA specimen in a petri-dish with the endocardium layer on the top, which was carefully removed. (**e**) LAA slices with 250 μm thickness were prepared using a vibratome. (**f**) The slices were placed on culture inserts, transferred into a 6-well plate filled with 1 ml medium and cultured via ALI method for 10 days.

Tissue preparation was performed under sterile conditions on a laminar flow bench using sterile instruments and sterile filtered solutions (Millipore vacuum filtration system). Due to interpatient morphological variability, about 70% of the obtained samples could be prepared. Samples unsuitable for dissection were not included in this study. Briefly, LAA was transferred into a petri dish filled with 4 $^\circ$C MTS. The appendage was cut open along the lateral side toward the apex of the tissue using curved iris scissors (Fig. 5a). The flat median area with sufficient muscle volume was dissected using microscissors and cut into $\sim$ 1 cm^2^ pieces (Fig. 5b,c). The rest of the tissue including trabeculae or any papillary muscles was disposed due to the extreme curvature as well as strong branching of these parts, which do not enable a proper vibratome slicing. The inner surface of the appendage is composed of relatively thick endocardium bearing a pronounced connective tissue layer, which can cause folding of the tissue during slicing leading to additional tissue damage^55^. Thus, the endocardium was carefully removed from dissected sections using razor blades or microscissors (Fig. 5d). The prepared tissue sections were mounted in 4% low melting agarose (dissolved in MTS) on a specimen holder. These tissue blocks were then glued with a histoacryl adhesive (B. Braun) epicardium-down to the sample plate and placed in the constantly cooled buffer tray filled with 4 $^\circ$C MTS. Before slicing, the blade position was improved by setting the height amplitude value to zero. The subsequent tissue sectioning was performed tangential to the exposed atrial myocardium in direction of the myofibrils upon continuous oxygenation with 0.05–0.10 mm/s blade advance rate, 2.00 mm amplitude and 250 μm slice thickness (Fig. 5e). The slices were collected in a tube filled with cold oxygenated MTS and stored for $\sim$ 0.5–1 h.

### Cultivation of atrial appendage slices

Based on protocol of Brandenburger et al. (2012)^21^, the slices were cultured via ALI method in medium 199 (Sigma Aldrich) supplemented with 1% penicillin/streptomycin and 1x ITS supplement (Insulin, Transferrin, Selenium; Sigma-Aldrich) at 37 $^\circ$C in humidified air with 5% CO$_2$. Prior to the culture, slices were washed in sterile PBS, placed on semi-porous tissue culture inserts (Millipore) and transferred in a 6-well culture plate filled with 1 ml cultivation medium (Fig. 5f). The medium was changed daily.

### Tissue metabolic activity

The overall metabolic activity of the ALI-cultured slices was investigated by methyl thiazolyl tetrazolium (MTT) assay (Abcam) at D0 (fresh), D1, D3, D5, D7 and D10 of cultivation. According to manufacturer’s instructions, the basic culture medium was exchanged with 1 ml MTT-containing culture medium (1:100 dilution). Following 4 h incubation at 37 $^\circ$C and 5% CO2, the slices were then directly placed in 600 μl MTT solvent reagent and left for incubation at 37 $^\circ$C and 5% CO2 overnight. The absorbance of supernatant was measured at 570 nm in a microtiter plate reader. The average values from triplicate readings per probe were adjusted by subtracting the average value for the blank value (MTT solvent reagent). The weight of wipe-dried tissue slice was determined using an analytical balance. The final metabolic activity value is given as measured optical density (OD) per weight unit (gram). Overall, 24 slices from 7 different patients were analyzed (4 slices per time point).

### Immunofluorescence staining

For super-resolution structured illumination microscopy (SIM), tissue slices were mounted in Tissue-Tek O.C.T. Compound on a specimen holder and frozen in liquid nitrogen. Three cryosections of 15 μm thickness representing top, middle and down layers were prepared at −20 $^\circ$C from one single slice using a cryostat (Leica). 24 slices obtained from 5 patients (4–6 slices from one biopsy) were frozen and sectioned at different time points of cultivation (D0, D1, D3, D5, D7 and D10). Thus, 12 sections were prepared per time point, which results in total 72 sections (3 sections per slice; 4 slices per time point; 6 time points). The cryosections were fixed with 2% paraformaldehyde for 10 min. After fixation, the sections were stained with 5 μg/ml wheat germ agglutinin Alexa Fluor 488 conjugate (Thermo Fisher) for 5 minutes. Following permeabilization with 0.1% Triton-X 100/TBS (Sigma Aldrich), protein block (DAKO) was applied for 20 min to prevent the non-specific background staining. Cardiac sarcomere network was labeled with primary mouse anti-sarcomeric $\alpha$-actinin antibody (dilution 1:200; ab9465, Abcam) overnight at 4 $^\circ$C. Secondary goat anti-mouse Alexa Fluor 647 antibody (dilution 1:350; A21237, Invitrogen) was applied next day for 2 h to visualize the $\alpha$-actinin. Finally, cryosections were mounted in Fluoroshield containing DAPI (Thermo Fisher) to stain cell nuclei.

### Super-resolution microscopy of sarcomere network and data analysis

The super-resolution microscopy and sarcomere network analysis was performed based on method described by Skorska et al.^19^. Briefly, images for SIM from three different microscopic fields per cryosection were recorded with the 40 ± alpha 1.46 plan apochromat objective with oil immersion using Zeiss LSM 780 confocal microscope equipped with Elyra PS.1 super-resolution system. Z-stacks were recorded in SIM mode with a 16 bit depth at 5 angles, with averaging 2; 23 μm grid was applied for 405 nm laser line (blue), 28 μm grid for 488 nm (green) and 51 μm grid for 633 nm (red). The acquired SIM dataset was reconstructed by the ZEN software (ZEN 2011 Sp7 FP3, LSM 7 Elyra, Version 14.0.0.0, Zeiss). Thus, 40 cardiomyocytes from overall of 36 microscopic fields (1-2 cells per microscopic field; 3 fields per section; 3 sections per slice; 4 slices per time point; 6 time points; 5 independent samples) were randomly selected for sarcomere analysis. Image analysis and evaluation of all parameters were conducted with Image J software. Sarcomere network density and particle size were determined by generation of binary images using Image J ridge detection plugin^56^. The filament density was calculated as percentage from the overall cell area. For detection of sarcomere orientation, microscopic images were aligned according to the longitudinal axis of the cell and orientation was quantified using directionality and orientation plugin for Image J^57–59^.

### Gene expression analysis by quantitative real-time PCR

Cultivated slices were nitrogen-frozen at time points D0 (fresh), D1, D3, D5, D7 and D10. Total RNA was purified from $\sim$ 20–30 mg nitrogen-frozen tissue using NucleoSpin RNA isolation kit (Macherey-Nagel) as suggested by manufacturer. High-Capacity cDNA Reverse Transcription Kit (Thermo Fisher) was used to synthesize complementary DNA (cDNA) with the following thermal cycler (Bio-Rad) program conditions: start with 10 min at 25 $^\circ$C, then temperature change to 37 $^\circ$C for 120 min following further increase to 85 $^\circ$C for 5 min and then cooling down to 4 $^\circ$C. The concentration of cDNA was spectrophotometrically measured by NanoDrop (Thermo Fisher). For the amplification reaction, 20 ng of cDNA template was mixed with following TaqMan gene expression assays (Thermo Fisher): GAPDH (Hs02786624_g1), ACTN2 (Hs00153809_m1), TNNT2 (HS00165960_m1), MYL2 (HS00166405_m1), MYL7 (HS1085598_g1), MYH6 (HS01101425_m1), CX43 (HS00748445_s1), KCJNA3 (Hs04334861_s1), RYR2 (HS00181461_m1). Subsequently, the samples were loaded on a StepOnePlus Real-Time PCR System (Applied Biosystems) and further proceeded upon standard experimental settings for 50 cycles (start at 50 $^\circ$C for 2 min, initial denaturation at 95 $^\circ$C for 10 min, denaturation at 95 $^\circ$C for 15 s and annealing/elongation at 60 $^\circ$C for 1 min). The real-time PCR data represented by CT values of target genes were normalized to corresponding endogenous control (GAPDH).

### Statistical analysis

All data are presented as mean ± standard error of the mean (SEM). Graphs demonstrate data from four independent experiments representing samples obtained from 7 different patients. For sarcomere network analysis, a total of 40 cells per time point were included into examination. For other experiment, total of 4 slices per time point was evaluated. Statistical significance was calculated using a one-way ANOVA followed by Tukey post hoc test for multiple comparisons. For directionality analysis, two-way ANOVA was applied. Probability levels considered as statistically significant were *p $\le$ 0.05, **p $\le$ 0.01, ***p $\le$ 0.001 and ****p $\le$ 0.0001. Calculations and graph analysis were performed using GraphPad Prism 8.0.1 software (GraphPad Prism, Inc., San Diego, CA, USA).

### Supplementary Information

Supplementary Information 1.

Supplementary Video S1.

Supplementary Video S2.

Supplementary Video S3.

## Data Availability

Datasets that have been generated in the current study are available upon reasonable request from the corresponding author.
