# Supplementary material for: Sarcomeric network analysis of ex vivo cultivated human atrial appendage tissue using super-resolution microscopy
Source: Sci Rep. 2023 Aug 10;13:13041. doi: 10.1038/s41598-023-39962-1 (PMC10415305; doi:10.1038/s41598-023-39962-1)
Supplement: Supplementary file 1 — Supplementary Information 1. [file 41598_2023_39962_MOESM1_ESM.pdf]

Supplementary material

Sarcomeric network analysis of *ex vivo* cultivated human atrial appendage tissue using super-resolution microscopy

Oleksandra Chabanovska, Heiko Lemcke, Hermann Lang, Brigitte Vollmar, Pascal M. Dohmen, Robert David*, Christian Etz, and Catharina Neßelmann

*robert.david@med.uni-rostock.de

Video legends:

Video 1: Suppvideo1_LAAslice_basal_d0.mp4

A representative bright-field microscopic recording demonstrates several regions of contracting cardiomyocytes (marked by black arrows) within a freshly prepared cardiac slice derived from left atrial appendage (LAA) immediately after cultivation start (D0) without electrical stimulation. Scala = 100 µm.

Video 2: Suppvideo2_LAAslice_basal_d3.mp4

A representative bright-field microscopic recording displays a region of contracting cardiomyocytes (marked by black arrows) within cultured LAA slice at D3 (72 h after cultivation start) without electrical stimulation. Scala = 100 µm.

Video 3: Suppvideo3_LAAslice_MTT_d5.mp4

A representative bright-field microscopic recording shows beating cultured LAA slice during metabolization of an MTT salt (MTT assay) at D5. Scala = 100 µm.

<u>*Note from authors*</u>: VLC video player is recommended to open the supplemented video files. Accessing the recording using other media player software might alter the video rendering resulting in an inaccurate playback.
